# Age-Specific Correlates of Child Growth

**DOI:** 10.1007/s13524-015-0449-3

**Published:** 2016-01-04

**Authors:** Matthias Rieger, Sofia Karina Trommlerová

**Affiliations:** International Institute of Social Studies (ISS), Erasmus University Rotterdam, Kortenaerkade 12, 2518 AX Den Haag, The Netherlands; Graduate Institute of International and Development Studies, Geneva, Switzerland

**Keywords:** Height-for-age, Child growth, Age heterogeneities, Gradient, Mother fixed effects

## Abstract

*Growth faltering* describes a widespread phenomenon that height- and weight-for-age of children in developing countries collapse rapidly in the first two years of life. We study age-specific correlates of child nutrition using Demographic and Health Surveys from 56 developing countries to shed light on the potential drivers of growth faltering. Applying nonparametric techniques and exploiting within-mother variation, we find that maternal and household factors predict best the observed shifts and bends in child nutrition age curves. The documented interaction between age and maternal characteristics further underlines the need not only to provide nutritional support during the first years of life but also to improve maternal conditions.

**Electronic supplementary material:**

The online version of this article (doi:10.1007/s13524-015-0449-3) contains supplementary material, which is available to authorized users.

## Introduction

Despite substantial improvements that have been achieved over the last 25 years, child malnutrition is still a pressing problem, mainly in South Asia and sub-Saharan Africa. In these two regions alone, 121 million or 38 % of children are stunted—that is, they are too small for their age (United Nations Children’s Fund 2013). Stunting in children does not occur uniformly over time: stunting rates have been shown to vary with age, both in cross-sectional and longitudinal data. In their seminal study, using cross-sectional data from 39 developing countries, Shrimpton et al. (2001) found a steeply decreasing height-for-age (HAZ, an indicator of stunting) for child cohorts between birth and age 2.^1^ They also observed that weight-for-age (WAZ) decreases substantially in age groups of children 3 to 12 months old. In response to these findings, several global health policy and information campaigns have been initiated (see Prentice et al. 2013).^2^ Despite being based on cross-sectional rather than longitudinal data, the literature coined the term “growth faltering” to describe these patterns (see, e.g., Allen 1994).

In this article, we discuss age-specific correlates of child growth—that is, factors that shift and bend the observed HAZ-age and WAZ-age profiles—in a sample of 56 developing countries. Because models investigating age patterns in child nutrition using cross-sectional data should be specified carefully (Cummins 2013), we introduce a new modeling approach: mother fixed effects with extensive controls for time trends. Thanks to this methodological improvement, we conjecture that our results could be interpreted as indicative of growth faltering.

Studying the drivers of growth faltering in children is crucial given the overwhelming adverse effects of poor nutritional status in early childhood on later-life outcomes. First and most important, undernutrition profoundly increases the vulnerability to disease and premature death. Worldwide, an estimated 3.1 million or 45 % of annual child deaths can be linked to undernutrition (Black et al. 2008). In the longer term, early childhood undernutrition impedes cognitive development (Mendez and Adair 1999), and the accumulation of human capital and educational achievement (Adair et al. 2013; Gandhi et al. 2011; Glewwe and Jacoby 1995; Glewwe et al. 2001; Maluccio et al. 2009; Victora et al. 2008). Finally, insufficient nutrition during childhood can lower economic success and productivity in the later stages of life (Hoddinott et al. 2008).

The main objective of this article is to identify age-specific correlates of child health that would point toward drivers of growth faltering. Previous studies have largely focused on age *patterns* of growth faltering, either across regions of the world using cross-sectional data (e.g., Prentice et al. 2013; Shrimpton et al. 2001) or in single countries for which longitudinal data were available (Maleta et al. 2003). Some studies also explored underlying age *heterogeneities* but only to a certain extent and in a limited sample (e.g., Sahn and Alderman 1997). We, on the other hand, not only explicitly model the nutrition-age profiles, but we also interact them with the “usual suspects” linked to low height- and weight-for-age in order to reveal age heterogeneities.^3^ Additionally, we investigate which group of factors (measured at country, community, and mother level) can best predict the observed age patterns.

From the empirical point of view, disentangling the determinants of child growth is not an easy task. A recent conceptual framework by Black et al. (2008) indicates that the underlying causes of household poverty are lack of various forms of capital (human, physical, financial) and of socioeconomic and political stability in countries and regions. And household poverty is, in turn, an immediate cause of poor nutritional status. In particular, poor households and their children are more vulnerable to diseases and food insecurity and are more prone to having poor hygiene and inadequate childcare and nutrition. In other words, the nutritional status is embedded in a complex system where many variables are not observable (e.g., parental ability) or not easily measurable (e.g., political stability).

Our proposed empirical strategy simplifies this system considerably by evaluating age heterogeneities *within* families. More specifically, we use a large household data set in which some mothers have more than one child under the age 5, and we compare the nutritional status of these children. By comparing siblings, we account for the main impact of both observable and unobservable characteristics of mothers, households, communities, and countries. Thus, we are able to identify age heterogeneities in child growth conditionally on the main effects of these factors.

The value added of our analysis compared against existing studies is thus sixfold. First, we examine *age-specific* correlates of child growth. Second, we model the profile of HAZ and WAZ as a function of age.^4^ We argue that both age profiles and age interactions should always be accounted for when modeling the determinants of child growth (see also Cummins 2013). Third, we explore a broad set of potential determinants of growth faltering in children, covering biological, socioeconomic, environmental, and macroeconomic factors. Fourth, we study which group of characteristics is the biggest predictor of child growth across age groups. Fifth, we find statistically and economically significant results when we control for mother fixed effects, a comprehensive set of time trends, and age interactions. Previous analyses have used only country or village fixed effects at best. Finally, our results can be regarded as more general because they are based on a large sample of 56 developing countries rather than on one particular country (e.g., Sahn and Alderman 1997). Apart from ensuring external validity of our findings, the large sample size is crucial for estimating flexible age curves and introducing age interactions and mother fixed effects.

Overall, our most important finding is that maternal and household characteristics—both observable and unobservable—are the best predictors of age patterns in child nutrition. For instance, the positive correlation between maternal education and child height or weight increases strongly with age, suggesting that maternal education has a cumulative impact on child health.

## Previous Research

In this section, we briefly review the relevant literature on (1) the main determinants of child nutrition, (2) growth faltering following the seminal study by Shrimpton et al. (2001), and (3) age heterogeneities in child growth determinants. We combine these three strands of research in a systematic study of age heterogeneities that can be interpreted as suggestive evidence of growth-faltering drivers for many countries.

First, our empirical model is related to a large literature on the determinants of child nutrition at the country, community, household, and child level. At the country level, macroeconomic growth has been found to benefit child nutrition (Haddad et al. 2003; Headey 2013; Heltberg 2009; Ravallion 1990; Smith and Haddad 2002).^5^ At the community level, a striking link to sanitation—in particular, open defecation—has been documented by Spears (2013) in India. Also, the importance of access to health services for better child nutrition has been frequently noted (e.g., Headey 2013). At the household level, much of the focus has been on maternal characteristics, such as literacy and education (see Bicego and Boerma 1993; Desai and Alva 1998; Headey 2013; Thomas and Strauss 1992; Thomas et al. 1991), maternal height (Monden and Smits 2009), and parity and smoking (Ong et al. 2002). Finally, risk factors at the child level include birth order (e.g., Rutstein 2005), preceding birth interval (Dewey and Cohen 2007), breast-feeding (Ong et al. 2002), and gender (Jayachandran and Pande 2015).

Second, our study builds on a number of descriptive studies on growth faltering in the spirit of Allen (1994) and Shrimpton et al. (2001). Victora et al. (2010) verified the Shrimpton et al. (2001) findings in a larger sample of 54 countries and using the new WHO growth standard (WHO 2006). As their main result, Victora et al. (2010) reported even more striking growth faltering patterns in the first 24 months, thereby confirming that the first two years of life are a “window of opportunity” for nutrition interventions. On a smaller scale, Maleta et al. (2003) used longitudinal rather than cross-sectional evidence from Malawi, finding that height and weight faltering occurs during the first 36 months and 3 to 12 months of life, respectively. Further nutrition-age curves were reported by Martorell and Young (2012) for India and Guatemala, by Engebretsen et al. (2008) for Uganda, and by Saha et al. (2009) based on longitudinal data covering the first 24 months of life of children in Bangladesh.

Third, with only a few exceptions, the literature on child health determinants in developing countries has not investigated age heterogeneities in a systematic way, possibly because of small sample sizes. Moreover, studies that did look at age heterogeneities did not include a comprehensive set of age interactions in their regression models. In a study on Mozambique, Sahn and Alderman (1997) split the sample into children younger than and older than 24 months of age and found that while maternal education is important for younger children, household income matters for the older group. Fernald et al. (2012) found that positive gradients associated with wealth and education tend to grow in importance over the first two years of life. Comparable wealth-age patterns also hold for a wide array of child development measures in data from Madagascar (see Fernald et al. 2011). Fotso et al. (2012:1) used longitudinal data from Kenya and reported that “assets poverty and subjective poverty have stronger relationships [. . .] with undernutrition at older age (24 months or older for assets poverty, and 12 months or older for subjective poverty).” More recently, Headey et al. (2015) tried to explain substantial improvements in nutritional status of Bangladeshi children using five rounds of Demographic and Health Surveys. Although the authors reported large upward shifts of the HAZ-age profile due to economic growth, the shape of the age profile has not changed, which indicates that growth faltering is still a problem. A study using Indonesian panel data by Cameron and Williams (2009) found that even though household poverty is associated with lower child health, this effect is not magnified as children age. This result is in line with evidence from England (Currie et al. 2007) but is in contrast with evidence from the United States and Canada, where health conditions of children from disadvantaged families do worsen as they age (Case et al. 2002; Currie and Stabile 2003). Finally, a few studies have examined health inequality (rather than child nutrition) across the whole age distribution. For instance, Diaz (2002) looked at chronic health problems, self-assessed health scores, and health expectancy in Brazilian children and adults, and concluded that “pro-rich inequalities increase with age” (p. 153).

To the best of our knowledge, our study is the first to systematically investigate age- specific correlates of child growth for a large array of covariates and countries. Whereas most studies have identified differential effects between two predefined age groups, we look across the whole age distribution of under-5 children. More importantly, we introduce a new mother fixed-effects model with a comprehensive set of time-trend controls and age interactions, thus coming closer to the identification of possible growth-faltering drivers.^6^ Additionally, we also aim at quantifying the set of covariates that best explains the observed age heterogeneities.

## Data

The data on children, their mothers, and household characteristics stem from the Demographic and Health Surveys (DHS). Our sample comprises 56 countries in which the DHS surveys were conducted between 2001 and 2013; we always take the most recent survey since 2000. A list of countries and sample sizes for each survey is available in Online Resource 1, Table S1.

Our sample includes all six world regions, as classified by the World Bank: sub-Saharan Africa (SSA; 31 countries), Latin America and the Caribbean (LAC; 8 countries), Europe and Central Asia (ECA; 7 countries), South Asia (SA; 5 countries), Middle East and North Africa (MENA; 3 countries), and East Asia and Pacific (EAP; 2 countries). The data contain information on 262,130 mothers along with their 350,152 children younger than 5 years. Table 1 shows descriptive statistics of all variables used in the analysis.

**Table 1:** Descriptive statistics of children

Variable	Full Sample	Observations	MFE Sample	Non-MFE Sample	Difference
1. Means of Dependent Variables					
Height-for-age *z* score	–1.51	305,978	–1.58	–1.45	–0.12
Weight-for-age *z* score	–1.28	316,400	–1.33	–1.23	–0.10
2. % of Children With Following Characteristics					
Female	48.6	323,720	49.8	47.4	2.4
Firstborn	26.6	323,720	17.8	35.1	–17.3
Second-born	24.6	323,720	27.3	22.0	5.3
Third- or later-born	48.8	323,720	54.8	42.9	11.9
Mother is short^a^	62.0	323,720	60.6	63.3	–2.7
Mother married young^a^	66.1	323,720	68.1	64.2	3.9
Mother has no education	42.5	323,720	47.8	37.4	10.4
Mother has primary education	24.0	323,720	24.5	23.5	1.0
Mother has secondary education	28.1	323,720	24.1	32.0	–7.9
Mother has tertiary education	5.4	323,720	3.6	7.1	–3.5
1st wealth quintile (the poorest)	23.8	323,720	26.7	21.0	5.7
2nd wealth quintile	21.9	323,720	22.9	20.9	2.0
3rd wealth quintile	20.2	323,720	20.4	19.9	**0.5**
4th wealth quintile	18.8	323,720	18.2	19.4	–1.2
5th wealth quintile (the richest)	15.3	323,720	11.7	18.7	–7.0
Rural area	71.9	323,720	75.5	68.4	7.1
Country suffered drought in survey year	8.6	323,720	9.3	7.9	1.4
Country had low under-5 mortality in survey year^a^	20.6	323,720	15.9	25.2	–9.3
Country had low GDP per capita in survey year^a^	43.2	323,720	42.7	43.7	*–1.0*
3. Means of Continuous and Underlying Explanatory Variables					
Child’s age in months	29.1	323,720	29.7	28.6	1.0
Birth order	3.1	323,720	3.3	2.8	0.5
Mother’s height in meters	1.5	323,720	1.5	1.5	0.0
Mother’s age at survey in years	27.6	323,720	27.5	27.8	–0.3
Mother’s age at marriage in years	17.6	323,720	17.5	17.8	–0.3
Country-level under-5 mortality per 1,000 live births	81.9	323,720	85.9	77.9	8.0
Country-level GDP per capita PPP in 2011 international dollars	3,762.8	323,720	3,637.5	3,884.7	–247.2

*Notes:* Descriptive statistics of underlying variables are shown in the third panel, “Means of continuous and underlying explanatory variables.” Column “Difference” refers to the difference between MFE and non-MFE sample; all differences are statistically significant at 1 % level with the exception of underlined italic differences (significant at 10 % level) and underlined bold differences (insignificant).

^a^Variable takes a value of 1 if the corresponding underlying variable is equal to or lower than the overall sample median, and 0 otherwise. Variable “country had low under-5 mortality in survey year” is an exception: it indicates that the child lives in a country with under‐5 mortality below the median in this sample of 56 countries.

The dependent variables in our analysis are the standard measures of nutritional status of children: *z* scores of height-for-age (HAZ) and weight-for-age (WAZ). Children in our final sample are, on average, 1.51 standard deviations shorter for their age than the median of the WHO child growth standard. In terms of weight-for-age, the average *z* score is –1.28: that is, these children are, on average, 1.28 standard deviations below the median weight in the reference population. Age ranges from 0 to 59 months, with an average of 29.1 months, and the age distribution is roughly uniform.^7^

Potential explanatory variables are measured at the child, mother, household, and country level. Children’s characteristics that are identified as potential determinants of child growth are gender, birth order, preceding birth interval, and breast-feeding. Mother’s characteristics of importance are her height, mortality of her children (to proxy the immediate health environment), age at first marriage, literacy and education, parity, and smoking.

Household characteristics of interest are differences in parental education (Thomas et al. 1990), household size (Lanjouw and Ravallion 1995), access to electricity (Thomas and Strauss 1992), access to sanitation (Spears 2013), access to health centers (Mani 2012; Schott et al. 2013), wealth (Fernald et al. 2012; Schott et al. 2013), and area of residence (Fink et al. 2014).^8^ Given that previous evidence has suggested that child health is linked to macroeconomic conditions, we use GDP per capita, country-level under-5 mortality and calories availability, and a drought indicator as a proxy of exogenous shocks (Hoddinott and Kinsey 2001). All variables are measured at the time of the survey.^9^

Similar to Shrimpton et al. (2001), we currently do not have comparable longitudinal data across many countries and regions of the world that would allow us to document growth faltering on a larger scale. Therefore, we use cross-sectional data in which children are observed only once, thus identifying age-specific correlates, heterogeneities across age groups, or cross-sectional dips in child growth rather than growth faltering per se.^10^ Nevertheless, we argue that our preferred mother fixed-effects specification with extensive controls for time trends is indeed pointing toward the drivers of growth faltering. At the same time, it is important to note that this specification reduces our sample to mothers with at least two children whose anthropometric measures were taken (i.e., children under age 5) because it compares children of different ages within a household.^11^

**Fig. 1:**
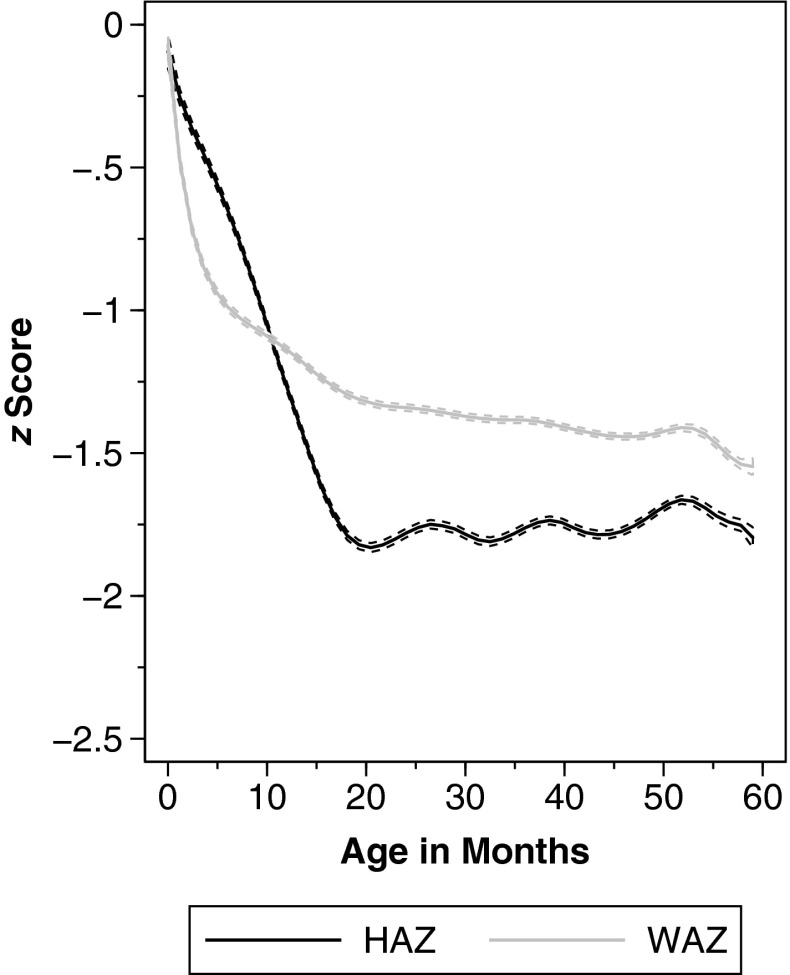
Anthropometric age profiles. Age profiles of height-for-age (HAZ) and weight-for-age (WAZ) *z* scores. Weighted local polynomial smooths. Dashed lines represent 95 % confidence intervals

Figure 1 underlines that age profiles differ substantially for HAZ and WAZ.^12^ Whereas HAZ decreases substantially in the first 21 months of life, it remains fairly stable afterward, with only minor fluctuations. WAZ, on the other hand, decreases smoothly, and the rate of decrease slows with age. Based on this difference in the observed patterns, the HAZ-age and WAZ-age profiles are modeled differently in our regression models: WAZ is specified as a logarithmic function of age, thus allowing the rate of decline to decrease with age, whereas HAZ is specified as a linear function of age with a structural break. The latter procedure enables estimating different slopes before and after a certain age threshold and is consistent with the observed pattern of a steep negative slope followed by a horizontal line. The age threshold was estimated at 21 months of life in the overall sample.^13^

## Descriptive Evidence on Age-Specific Correlates of Child Growth

Before moving to the regression analysis, we offer descriptive evidence on the cross-sectional age profiles of HAZ and WAZ. First, we explore regional patterns and relate them to stages of economic development. Afterward, we decompose age profiles by demographic characteristics and socioeconomic conditions.

### Cross-Sectional Age Profiles Across Regions and Stages of Economic Development

**Fig. 2:**
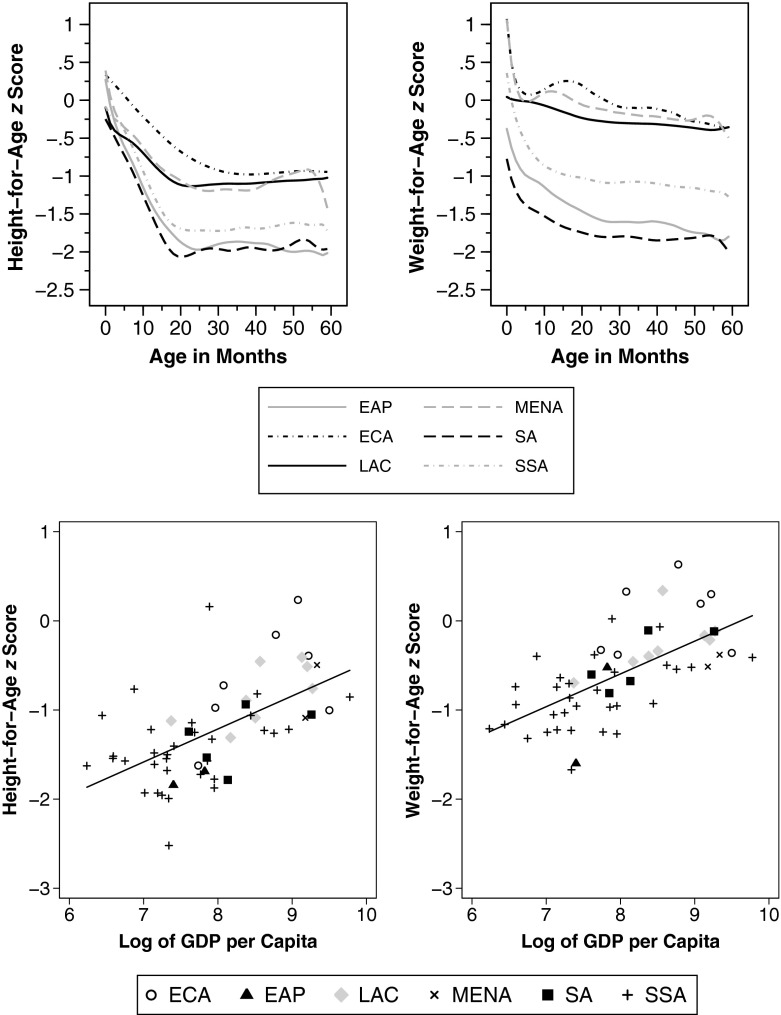
Anthropometric age profiles by regions and stages of economic development. The upper panel shows weighted local polynomial smooths in six regions of the developing world. The lower panel shows age group differences by country and region, measured as the difference in weighted average height-for-age (or weight-for-age) *z* score in the country between children aged 21–24 months and children aged 0–3 months. The line represents a linear fit. Regions are abbreviated as follows: East Asia and Pacific (EAP), Europe and Central Asia (ECA), Latin America and the Caribbean (LAC), Middle East and North Africa (MENA), South Asia (SA), sub-Saharan Africa (SSA)

There are substantial differences in age curves across regions of the world, as illustrated in the top panel of Fig. 2. Although height-for-age decreases steeply up to the age group of roughly 21 months in all regions (except for ECA, up to 35 months), the extent of this dip differs greatly: whereas older age groups in MENA, LAC, and ECA regions exhibit smaller declines, the dips are significantly more pronounced in SSA and South and East Asia.^14^ Interestingly, SSA is economically worse off than both East (EAP) and South Asia (SA) but better off in terms of HAZ and WAZ. There are two debated explanations for this pattern—(1) genetic differences and (2) different social preferences (in particular, the eldest son preference in parts of Asia). A recent study by Jayachandran and Pande (2015) showed that male firstborns in India tend to be *taller* than male firstborns in SSA, whereas the later-born Indian children are shorter than the firstborn son *and* than their counterparts in SSA. And indeed, a closer look into our data suggests that son preference could explain the observed regional differences in age curves.^15^ Nevertheless, we cannot exclude the possibility that genetics also matters—for instance, through differential growth paths or different propensity to respond to environmental conditions.

Because the world regions differ in their stages of economic development, we now examine to which extent the severity of cross-sectional dips in age-profiles is associated with a country’s GDP. In the bottom panel of Fig. 2, we plot country-level changes in *z* scores between age groups 0–3 and 21–24 months against economic conditions. We find less pronounced dips when GDP per capita is high. In other words, economic development is associated with flatter age curves. When we fit a linear regression line, the difference in the predicted HAZ dip between the richest and the poorest country amounts to 1.31; the corresponding dip for WAZ is 1.30.

### Child Growth Age Profiles by Demographic and Socioeconomic Characteristics

**Fig. 3:**
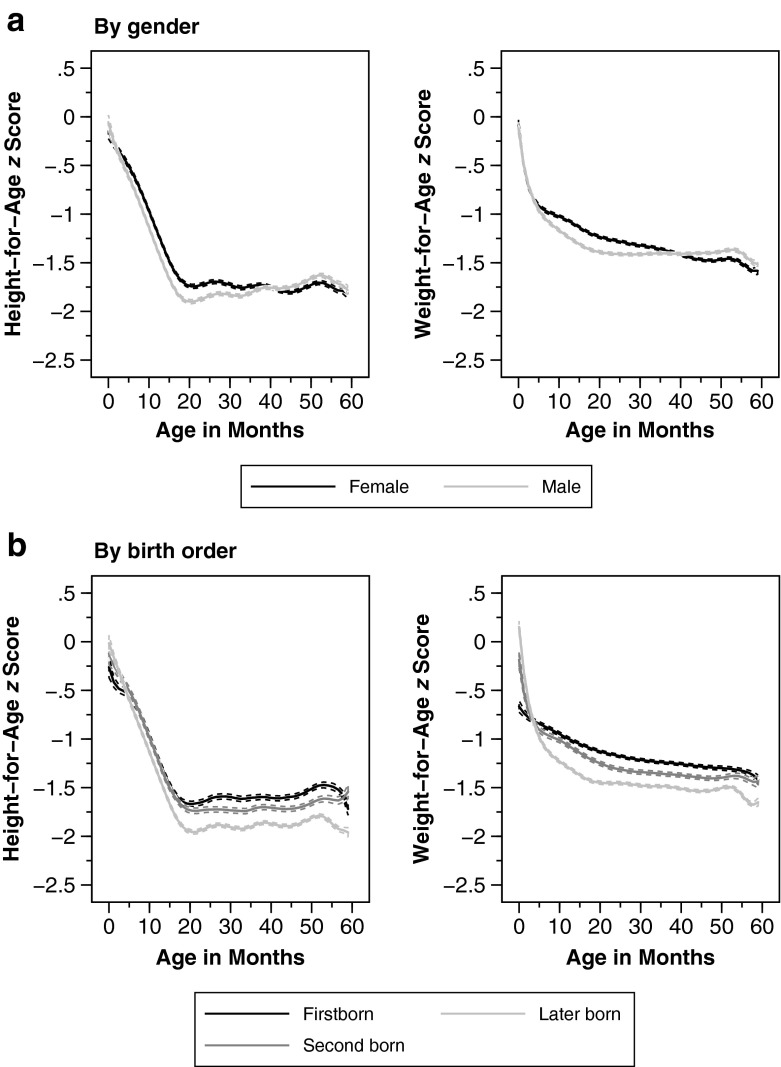
Anthropometric age profiles by children’s characteristics. Age profiles of height-for-age and weight-for-age *z* scores by gender and birth order. Weighted local polynomial smooths. Dashed lines represent 95 % confidence intervals

**Fig. 4:**
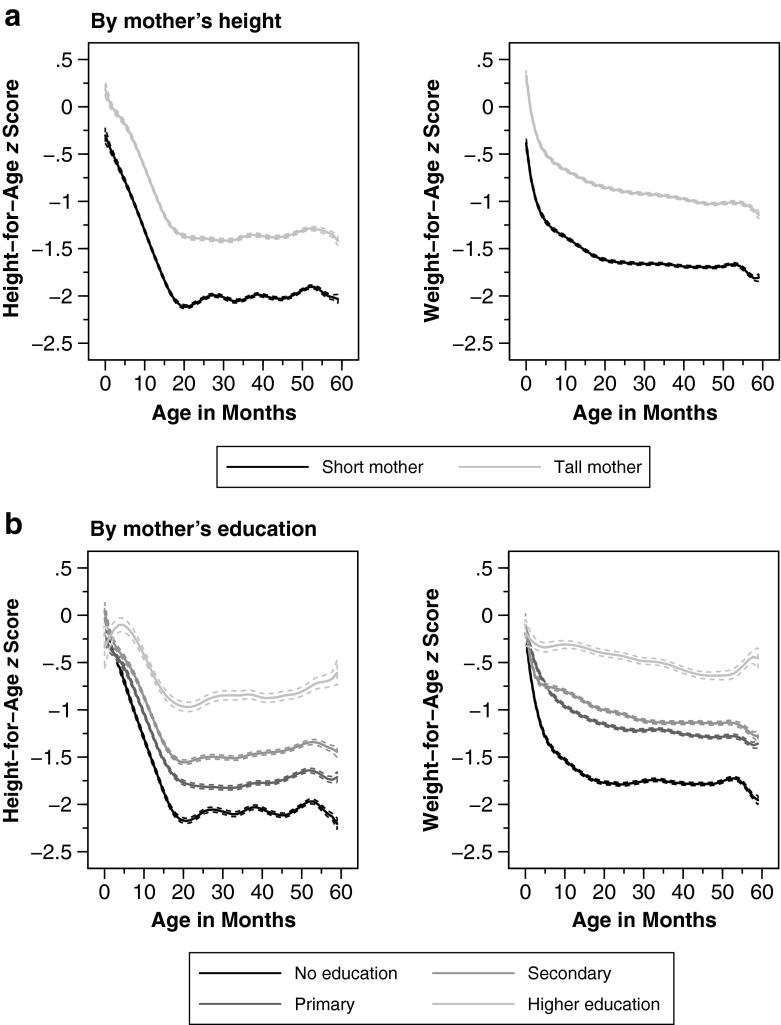
Anthropometric age profiles by mother’s characteristics. Age profiles of height-for-age and weight-for-age *z* scores by maternal height and education. Weighted local polynomial smooths. Dashed lines represent 95 % confidence intervals

**Fig. 5:**
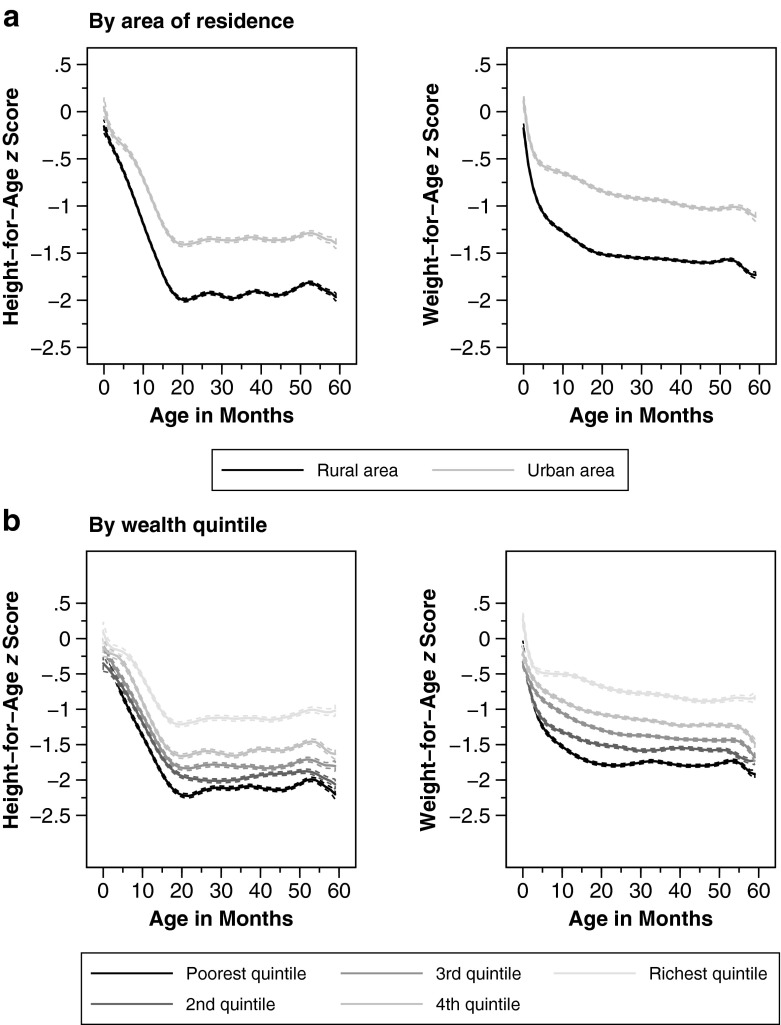
Anthropometric age profiles by household characteristics. Age profiles of height-for-age and weight-for-age *z* scores by residence (rural vs. urban) and wealth quintile. Weighted local polynomial smooths. Dashed lines represent 95 % confidence intervals

Apart from macroeconomic conditions, the nutrition-age curves may also differ by demographic and socioeconomic characteristics. In what follows, we graph age curves by commonly used determinants of nutritional status at the child (Fig. 3), mother (Fig. 4), and household level (Fig. 5).

First, do age curves vary by gender and birth order? The top panel of Fig. 3 reports no sizable differences for female and male children across age groups. On the other hand, we do see important and long-lasting differences when considering birth order in the bottom panel of Fig. 3. Although HAZ of young cohorts does not differ by birth order, later-born children are noticeably worse off among older cohorts. Interestingly, this birth order difference is found also for weight (WAZ) of older cohorts, even though the firstborn children have considerably *lower* weight at birth.

Second, can we observe age heterogeneities by maternal characteristics, such as mother’s height and education? According to the top panel of Fig. 4, children of tall mothers are taller and heavier already at birth, and this difference is not substantially higher for older age cohorts. On the other hand, maternal education, an often-cited strong correlate of child growth, is associated with a substantively smaller dip in HAZ and WAZ (bottom panel of Fig. 4).

Finally, Fig. 5 displays how cross-sectional correlation between child growth and age groups varies by area of residence and household wealth. Both correlates affect the shape of age curves: children living in rural areas and in households with lower wealth (as measured by wealth quintile) exhibit bigger dips in both child height and weight.

## Regression Model

So far, we have documented important age heterogeneities with respect to observable predictors of child nutrition in a bivariate framework. However, it is unclear how robust these patterns are to accounting for a wide array of covariates, time trends, and unobservable factors. To eliminate the latter, we propose to use a mother fixed-effects model that compares children of different ages born to the same mother. The biggest advantage of this approach is that it enables us to control not only for observable variables, such as mother’s education and household infrastructure, but also—and more importantly—for mother-, household-, and country-level unobservable characteristics, such as maternal ability, social status of the household, and political stability in the country. Furthermore, we introduce interactions between the age profile and characteristics of interest in order to verify how, say, maternal factors such as education and height correlate with height-for-age across different age groups.

In order to model interactions between demographic and socioeconomic characteristics and height-for-age (*haz*) age profile of child *k*, born to mother *m*, in household *h*, country *c*, and region *r*, we specify the following model:

1 $$ \begin{array}{l} ha{z}_{kmhcr}= constant+ child\  age\  in\  month{s}_{kmhcr}\times {\mathbf{R}}_r^{\mathbf{\prime}}{\boldsymbol{\upbeta}}_1+ structural\  brea{k}_{kmhcr}\times {\mathbf{R}}_r^{\mathbf{\prime}}{\boldsymbol{\upbeta}}_2+{\left(\begin{array}{c}\hfill {\mathbf{K}}_{kmhcr}\hfill \\ {}\hfill {\mathbf{M}}_{mhcr}\hfill \\ {}\hfill {\mathbf{H}}_{hcr}\hfill \\ {}\hfill {\mathbf{C}}_{cr}\hfill \end{array}\right)}^{\prime }{\boldsymbol{\upbeta}}_3\hfill \\ {} + child\  age\  in\  month{s}_{kmhcr}\times {\left(\begin{array}{c}\hfill {\mathbf{K}}_{kmhcr}\hfill \\ {}\hfill {\mathbf{M}}_{mhcr}\hfill \\ {}\hfill {\mathbf{H}}_{hcr}\hfill \\ {}\hfill {\mathbf{C}}_{cr}\hfill \end{array}\right)}^{\prime }{\boldsymbol{\upbeta}}_4+ structural\  brea{k}_{kmhcr}\times {\left(\begin{array}{c}\hfill {\mathbf{K}}_{kmhcr}\hfill \\ {}\hfill {\mathbf{M}}_{mhcr}\hfill \\ {}\hfill {\mathbf{H}}_{hcr}\hfill \\ {}\hfill {\mathbf{C}}_{cr}\hfill \end{array}\right)}^{\prime }{\boldsymbol{\upbeta}}_5\hfill \\ {} + child\  age\  in\  month{s}_{kmhcr}\times structural\  brea{k}_{kmhcr}\times {\left(\begin{array}{c}\hfill {\mathbf{K}}_{kmhcr}\hfill \\ {}\hfill {\mathbf{M}}_{mhcr}\hfill \\ {}\hfill {\mathbf{H}}_{hcr}\hfill \\ {}\hfill {\mathbf{C}}_{cr}\hfill \end{array}\right)}^{\prime }{\boldsymbol{\upbeta}}_6\hfill \\ {} + child\  age\  in\  month{s}_{kmhcr}\times structural\  brea{k}_{kmhcr}\times {\mathbf{R}}_{\mathbf{r}}^{\mathbf{\prime}}{\boldsymbol{\upbeta}}_7+{\upvarepsilon}_{kmhcr},\hfill \end{array} $$

where the coefficient vector **β**_1_ captures the main association between HAZ and child’s age in months. The literature has found this coefficient to be negative (see Victora et al. 2010). As discussed in the Data section, we model the kink in the HAZ-age curve with the dummy variable *structural break*, which varies by region.^16^ Additionally, the entire HAZ-age profile is allowed to differ by region through an interaction with **R**_*r*_  , a matrix of regional dummy variables. The matrices **K**, **M**, **H**, and **C** collect characteristics that vary at the level of the child (child’s gender, birth order), mother (mother’s height, age at marriage, education), household (wealth quintile, area of residence), and country (drought, under-5 mortality, and GDP per capita in the survey year), respectively. We also add year-of-survey fixed effects to control for the 2001–2013 time span between the surveys. We present regression results based on 10 of 17 potential explanatory variables; five additional variables are considered in subsample analyses. The final set of variables is selected based on two criteria: (1) the variable exhibits significant interaction terms in a univariate regression explaining either HAZ or WAZ, and (2) the interaction terms of the selected variables remain significant also in the full regression on either HAZ or WAZ.^17^

Because the main purpose of this article is to estimate how demographic and socioeconomic characteristics bend the nutrition-age curve, we interact child age in months with the observable characteristics **K**, **M**, **H**, and **C**. Vector **β**_4_ captures to which extent the age profile bends in young age, before the structural break occurs. Similarly, we interact the variables with the structural break itself, storing the associated coefficients in the vector **β**_5_. Furthermore, we add two additional interaction terms to complete the model.

So far, Eq. (1) represents a simple ordinary least squares (OLS) model in which many relevant factors might be unobserved and the coefficients might be therefore biased. In order to account for unobservable characteristics (measured at mother or any higher level) that determine child height, we decompose the error term in Eq. (1) into two parts:

2 $$ {\upvarepsilon}_{kmhcr}={\upsilon}_{mhcr}+{\vartheta}_{kmhcr}, $$

where *υ*_*mhcr*_ represents a mother fixed effect (MFE).^18^ Estimation of Eq. (1) with the error term as specified in Eq. (2) corresponds to a “within-mother” transformation after which all mother-, household-, and country-specific variables **M**, **H**, and **C**, and year-of-survey fixed effects drop out:

3 $$ \begin{array}{l} ha{z}_{kmhcr}= child\  age\  in\  month{s}_{kmhcr}\times {\mathbf{R}}_r^{\mathbf{\prime}}{\boldsymbol{\upbeta}}_1+ structural\  brea{k}_{kmhcr}\times {\mathbf{R}}_r^{\mathbf{\prime}}{\boldsymbol{\upbeta}}_2+{\mathbf{K}}_{kmhcr}^{\mathbf{\prime}}{\boldsymbol{\upbeta}}_3\hfill \\ {} + child\  age\  in\  month{s}_{kmhcr}\times {\left(\begin{array}{c}\hfill {\mathbf{K}}_{kmhcr}\hfill \\ {}\hfill {\mathbf{M}}_{mhcr}\hfill \\ {}\hfill {\mathbf{H}}_{hcr}\hfill \\ {}\hfill {\mathbf{C}}_{cr}\hfill \end{array}\right)}^{\prime }{\boldsymbol{\upbeta}}_4+ structural\  brea{k}_{kmhcr}\times {\left(\begin{array}{c}\hfill {\mathbf{K}}_{kmhcr}\hfill \\ {}\hfill {\mathbf{M}}_{mhcr}\hfill \\ {}\hfill {\mathbf{H}}_{hcr}\hfill \\ {}\hfill {\mathbf{C}}_{cr}\hfill \end{array}\right)}^{\prime }{\boldsymbol{\upbeta}}_5\hfill \\ {} + child\  age\  in\  month{s}_{kmhcr}\times structural\  brea{k}_{kmhcr}\times {\left(\begin{array}{c}\hfill {\mathbf{K}}_{kmhcr}\hfill \\ {}\hfill {\mathbf{M}}_{mhcr}\hfill \\ {}\hfill {\mathbf{H}}_{hcr}\hfill \\ {}\hfill {\mathbf{C}}_{cr}\hfill \end{array}\right)}^{\prime }{\boldsymbol{\upbeta}}_6\hfill \\ {} + child\  age\  in\  month{s}_{kmhcr}\times structural\  brea{k}_{kmhcr}\times {\mathbf{R}}_{\mathbf{r}}^{\mathbf{\prime}}{\boldsymbol{\upbeta}}_7+{\upsilon}_{mhcr}+{\vartheta}_{kmhcr}.\hfill \end{array} $$

Note that even though the fourth term in Eq. (1) is reduced to **K**_*kmhc*_^′^**β**_3_, we are still able to identify the coefficients of interest **β**_4_ and **β**_5_ for all variables **K**, **M**, **H**, and **C**.

One limitation of our study is that we are using cross-sectional rather than longitudinal data to investigate age-specific correlations. To eliminate as many potential confounding factors as possible, we net out time trends and seasonalities that may overlap with the age group comparisons by adding (1) country-specific linear time trends in terms of year of birth, (2) year-of-birth fixed effects, (3) 12 calendar-month-of-birth fixed effects, and (4) 12 difference-between-calendar-month-of-survey-and-calendar-month-of-birth fixed effects. Year-of-survey and month-of-survey fixed effects are already captured by the MFE.

Although we focus primarily on results for height-for-age, we also present complementary evidence using weight-for-age (*waz*) as a dependent variable. The corresponding semi-logarithmic model after the “within-mother” transformation is specified as follows^19^:

4 $$ \begin{array}{l}wa{z}_{kmhcr}={\boldsymbol{\upbeta}}_1 log\left( child\  age\  in\  month{s}_{kmhcr}\right)+{\mathbf{K}}_{kmhcr}^{\mathbf{\prime}}{\boldsymbol{\upbeta}}_2\hfill \\ {} + \log \left( child\  age\  in\  month{s}_{kmhcr}\right)\times {\left(\begin{array}{c}\hfill {\mathbf{K}}_{kmhcr}\hfill \\ {}\hfill {\mathbf{M}}_{mhcr}\hfill \\ {}\hfill {\mathbf{H}}_{hcr}\hfill \\ {}\hfill {\mathbf{C}}_{cr}\hfill \end{array}\right)}^{\prime }{\boldsymbol{\upbeta}}_3+{\upsilon}_{mhcr}+{\vartheta}_{kmhcr}.\hfill \end{array} $$

Finally, note that we weight the regressions with population-size-adjusted sampling weights and cluster standard errors at the primary sampling unit (i.e., cluster) level.

## Results

Which cross-sectional child growth correlates can be associated with the observed dips, bends, and breaks in the age curves in Figs. 1–5? In what follows, we present two sets of regression models identifying age-specific correlates of child health: a simple OLS model (as in Eq. (1)) and an MFE model (Eqs. (2)–(4)). We present both the main effects of the variables of interest and their interactions with the age profile. The left panel of Table 2 summarizes the OLS model for height-for-age. The first column presents the main correlation between our variables and height-for-age before the structural break (β_3_), whereas the third column shows this correlation after the structural break—that is, for the older age group (β_5_). Intuitively, β_3_ and β_5_ represent an upward or downward shift of the age curve before and after the structural break, respectively. The second column, on the other hand, lists the interactive effects between each variable and age before the structural break (β_4_), thus modeling the *bend* of the age profile for younger cohorts. The right panel of Table 2 shows the same set of coefficients estimated in the MFE model.^20^

**Table 2:** Regression results for height-for-age (HAZ)

	OLS			MFE		
Variable	Variable	Age × Variable	Break × Variable	Variable	Age × Variable	Break × Variable
Child Is a Girl^a^	0.081*	0.005^†^	0.263*	0.134*	–0.003	0.209*
	(0.037)	(0.003)	(0.060)	(0.061)	(0.005)	(0.099)
Child’s Birth Order	0.017^†^	–0.002*	–0.020	–0.910*	–0.001	–0.004
	(0.010)	(0.001)	(0.015)	(0.043)	(0.001)	(0.021)
Mother’s Height (in 10cm)	0.429*	0.005*	–0.016	––	0.009*	0.063
	(0.029)	(0.002)	(0.046)		(0.004)	(0.064)
Mother’s Age at Marriage	–0.003	0.001*	0.018*	––	0.002*	0.031*
	(0.005)	(0.000)	(0.008)		(0.001)	(0.012)
Mother’s Education (in levels)	0.065*	0.005*	0.069^†^	––	0.008*	0.151*
	(0.024)	(0.002)	(0.039)		(0.003)	(0.058)
Poorest Quintile^a^	–0.027	–0.011*	–0.129^†^	––	–0.016*	–0.103
	(0.050)	(0.004)	(0.078)		(0.006)	(0.103)
Rural Area^a^	–0.081^†^	–0.006*	–0.210*	––	–0.006	–0.232*
	(0.045)	(0.003)	(0.069)		(0.006)	(0.103)
Drought^a^	0.227*	–0.016*	–0.367*	––	–0.004	–0.621*
	(0.063)	(0.005)	(0.099)		(0.012)	(0.149)
Under-5 Mortality (per 100 births)	–0.001	–0.002*	–0.010	––	–0.006*	–0.036*
	(0.006)	(0.001)	(0.009)		(0.001)	(0.015)
GDP per capita PPP (in $1,000)	–0.009	0.002*	0.057*	––	0.004*	0.072*
	(0.006)	(0.000)	(0.014)		(0.001)	(0.024)
Number of Observations	305,967			305,967		
Observations Contributing to Variance	305,967			144,432		
*R* ^2^	.175			.245		

*Notes:* OLS and mother fixed effects (MFE) estimations are shown. Dependent variable is *z* score of height-for-age. Mother’s education is measured in four levels: no education, primary, secondary, and higher education. Country-level variables of drought, under-5 mortality, and GDP are measured in the year of the survey. “Break” is a region-specific dummy variable that takes a value of 1 if the child is older than 18 (SSA region), 21 (MENA, SA regions), 22 (EAP region), 23 (LAC region), and 35 (ECA region) months old; and 0 otherwise. Coefficients of age, “break,” “age × break,” their interactions with regional dummy variables (to allow for the main HAZ-age profile to vary by region), “age × break × variable,” year-of-survey fixed effects, and a constant are estimated but not shown. MFE specification also includes country-specific time trends, year-of-birth fixed effects, calendar-month-of-birth fixed effects, and difference-between-calendar-month-of-survey-and-calendar-month-of-birth fixed effects. Coefficients of variables that do not vary among siblings cannot be estimated in MFE specification. Regressions are weighted using sampling weights that adjust for population size. Standard errors are clustered at the primary sampling unit (cluster) level and are shown in parentheses.

^a^Binary variable.

^†^ *p* < .10; *p < .05

### Height-for-Age OLS Results

The OLS model in Table 2 (column 1) suggests that height-for-age is correlated with being a female child (+), birth order (+), maternal height (+) and education (+), rural residence (–), and drought shocks (+) in the first 21 months of life.

The main coefficients of interest measuring differential effects of variables by age are displayed in the second column. All demographic and socioeconomic variables interacted with age are significant—they are important correlates in child growth models. Some variables are associated with a pull away from, while others are correlated with a push toward, the median child in the healthy reference population. Pull factors (or negative correlations) are found for birth order, the lowest wealth quintile, rural households, and under-5 mortality and drought shocks at the time of the survey. Push factors (or positive correlations) are detected for being a female child; maternal height, education, and age at marriage; and GDP per capita. Column 3 indicates that most of these bends (push or pull) before the structural break lead to a long-lasting upward or downward shift (level effect) of the age profile afterward. In particular, signs of significant coefficients in column 3 go in the same direction as the age interactions in column 2.

Note that some of the main effects in column 1 are insignificant and/or of counterintuitive sign (e.g., drought, GDP per capita). Most likely, the naïve OLS estimates are capturing omitted structural characteristics across countries, communities, and households, which in turn bias the coefficients. For instance, drought at the time of the survey is, counterintuitively, positively associated with HAZ (β_3_). However, countries vulnerable to drought are likely to be systematically different in terms of time-invariant factors. Therefore, a better comparison would consider children in the same household and examine how the same drought shock affects them as a function of their age (see the upcoming MFE models). Nevertheless, even the simple OLS model illustrates that age structure is important when explaining cross-sectional correlates of child growth because the interactions of drought with age and the structural break variable in columns 2 and 3 are significant and negative.

### Height-for-Age MFE Results

The MFE model identifies age heterogeneities within families, thus addressing some of the problems of unobserved heterogeneity. As shown in the right panel of Table 2, column 5, six age interactions are significant and sizable in magnitude: maternal height (+), education (+), and age at marriage (+), as well as the poorest wealth quintile (–), under-5 mortality (–), and GDP per capita (+). Most of these variables bend the age profile so strongly that they lead to a significant long-lasting shift for older cohorts (column 6).

Notably, the effects of the country-level factors are more sizable in the MFE than in the OLS model. Drought is significantly associated with lower height-for-age among the age groups past the structural break, which occurs around 21 months of age. The effect is roughly double the size of the naïve OLS estimates, indicating omitted variable bias in the OLS. The measure of local disease environment, or under-5 mortality, is also strongly correlated with “pulling” children away from the healthy growth curve as they age.

Having identified the main age heterogeneities in the correlates of height, the next step is to establish their importance (i.e., the magnitudes of the estimated age effects). In what follows, we focus on variables that display significant interactions with age and the structural break in the MFE model. For example, the coefficient associated with maternal education (0.008) implies that going from no education to elementary education is associated with an increase in HAZ by 0.048 for a 6-month-old, compared with 0.096 for a 12-month-old baby in the same household.^21^ These effect sizes correspond to 3 % and 6 % of the sample mean in HAZ, respectively. In addition, because of this strong divergence in the first 21 months, the upward *shift* in the age curves associated with each level of maternal education is 0.151 after the structural break (see column 6 in Table 2). This additional level effect (on top of the unidentified main effect of education) corresponds to 10 % of the sample mean for each level of education, which amounts to 40 % of the average HAZ when comparing mothers with no versus tertiary education.

Apart from maternal characteristics, country-level variables also matter. In countries where under-5 mortality is 1 percentage point higher (i.e., an increase of 10 deaths per 1,000 live births), the HAZ is 0.036 *z* scores lower for every six months. This six-month decrease corresponds to 2.5 % of the sample mean of height-for-age.

Other variables that have been shown to be important for child growth and “growth faltering” are available for only a smaller subset of the data. These include access to health services (Mani 2012; Schott et al. 2013) and maternal or uterine factors (Ong et al. 2002). In an additional analysis (Tables S4 and S5 in Online Resource 1), we find only weak or insignificant correlations with access to health services, maternal smoking, breast-feeding, and measures of calories availability at the country level. However, we do find that the correlation of birth interval and child growth magnifies as we move up the age ladder.

### Weight-for-Age Results

In the MFE specification of the WAZ model (Table 3, columns 5 and 6), we find similarly striking age heterogeneities: three child growth correlates are associated with pulls away from (being a girl, drought shocks, and under-5 mortality) and three with pushes toward (mother’s age at marriage and education, and GDP per capita) the “healthy” growth curve as we climb up the age ladder. Again, the interaction term between maternal education and child’s age in months is sizable and significant: children born to mothers with higher educational level (e.g., secondary vs. primary education) gain by 0.072 *z* scores more in their height when moving up the age curve from 0 to 10 months than children of less-educated mothers. This effect corresponds to a substantial 6 % of the sample mean in weight-for-age. In years of drought, the loss in *z* score is 0.28, or 22 % of the sample mean.

**Table 3:** Regression results for weight-for-age (WAZ)

	OLS		OLS, MFE Sample		MFE	
Variable	Variable	Log(Age) × Variable	Variable	Log(Age) × Variable	Variable	Log(Age) × Variable
Child Is a Girl^a^	0.226*	–0.062*	0.211*	–0.055*	0.228*	–0.063*
	(0.035)	(0.010)	(0.050)	(0.015)	(0.053)	(0.016)
Child’s Birth Order	0.019*	–0.009*	0.023^†^	–0.013*	–0.648*	–0.001
	(0.009)	(0.003)	(0.013)	(0.004)	(0.030)	(0.004)
Mother’s Height (in 10cm)	0.423*	–0.029*	0.386*	–0.019^†^	––	0.017
	(0.026)	(0.008)	(0.036)	(0.011)		(0.011)
Mother’s Age at Marriage	0.003	0.005*	0.005	0.003	––	0.005*
	(0.005)	(0.001)	(0.007)	(0.002)		(0.002)
Mother’s Education (in levels)	0.142*	0.013^†^	0.146*	0.006	––	0.030*
	(0.023)	(0.007)	(0.033)	(0.010)		(0.010)
Poorest Quintile^a^	–0.078	–0.040*	–0.128*	–0.021	––	0.002
	(0.048)	(0.014)	(0.064)	(0.018)		(0.019)
Rural Area^a^	–0.126*	–0.009	–0.084	–0.015	––	–0.024
	(0.044)	(0.013)	(0.062)	(0.018)		(0.018)
Drought^a^	0.571*	–0.168*	0.562*	–0.155*	––	–0.117*
	(0.056)	(0.017)	(0.073)	(0.021)		(0.054)
Under-5 Mortality (per 100 births)	0.013*	–0.011*	0.026*	–0.014*	––	–0.044*
	(0.005)	(0.002)	(0.007)	(0.002)		(0.006)
GDP per capita PPP (in $1,000)	0.079*	–0.013*	0.092*	–0.015*	––	0.017^†^
	(0.007)	(0.002)	(0.009)	(0.003)		(0.009)
Number of Observations	316,389		316,389		316,389	
Observations Contributing to Variance	316,389		155,014		155,014	
*R* ^2^	.249		.229		.126	

*Notes:* OLS and mother–fixed effects (MFE) estimations are shown. Dependent variable is *z* score of weight-for-age. Mother’s education is measured in four levels: no education, primary, secondary, and higher education. Country-level variables of drought, under-5 mortality, and GDP are measured in the year of the survey. Coefficient of logarithm of age, its interaction with regional dummy variables (to allow for the main WAZ age profile to vary by region), year-of-survey fixed effects, and a constant are estimated but not shown. MFE specification also includes also country-specific time trends, year-of-birth fixed effects, calendar-month-of-birth fixed effects, and difference-between-calendar-month-of-survey-and-calendar-month-of-birth fixed effects. Coefficients of variables that do not vary among siblings cannot be estimated in MFE specification. Regressions are weighted using sampling weights that adjust for population size. Standard errors are clustered at the primary sampling unit (cluster) level and are shown in parentheses.

^a^Binary variable.

^†^ *p* < .10; *p < .05

To sum, maternal education and age at marriage as well as country variables (drought, GDP per capita, and under-5 mortality) are important age-specific correlates of child growth in terms of both height and weight. Some of these variables were identified as strong correlates of child health also in studies that do not take into account age-specific effects. Our results underline that age heterogeneities in these correlations should be accounted for in regression models of child nutrition. In other words, there is evidence of important health gradients.

### Sample Selection and Representativeness

Our preferred MFE model relies on within-mother variation, which is available only for mothers with more than one child in the sample. On the one hand, the MFE estimation has strong internal validity compared with OLS. On the other hand, the external validity, or “representativeness,” of the sample is slightly different, but not dramatically so. Table 1 shows averages of the main variables in the full and reduced sample. Because of the large sample size, differences in averages are statistically significant but often small in magnitude. Overall, one can say that children in the MFE sample are on average slightly worse off in terms of nutrition and maternal, household, and country characteristics.

Nevertheless, our ultimate goal is the “representativeness” of the estimated coefficients, even if the sample differs along some dimensions. In the WAZ estimation (Table 2), we find stable and qualitatively similar coefficients in OLS estimations from the full and the restricted sample; the magnitudes vary slightly.^22^ For instance, the coefficients on maternal education are 0.142 in the full sample and 0.146 in the reduced sample, despite the more substantial difference in the average values (shown in Table 1). The corresponding age-education interaction is less pronounced and more imprecise in the reduced sample using OLS (but not when mother fixed effects are introduced). To take into account these variations in coefficients, we interpreted them relative to the sample mean of the dependent variables.

## Grouping Age-Specific Correlates of Child Growth

**Fig. 6:**
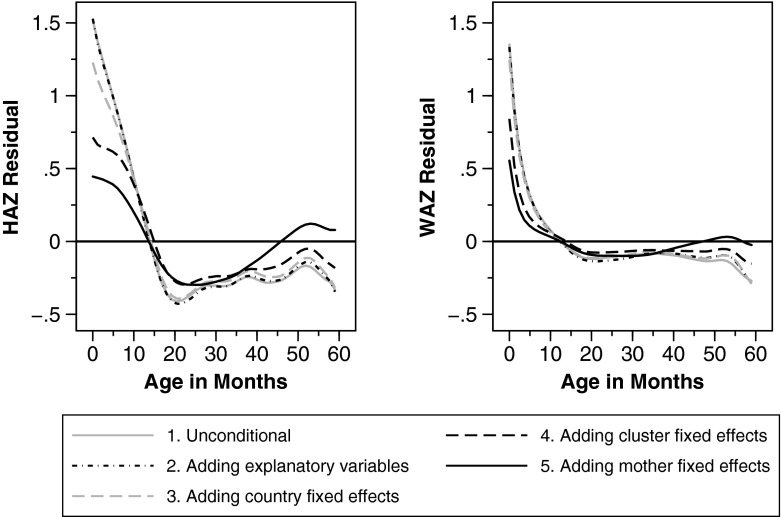
Residuals of anthropometric *z* scores. Residuals of height-for-age (HAZ) and weight-for-age (WAZ) *z* scores. Weighted local polynomial smooths of residuals from weighted regressions of HAZ and WAZ on five sets of variables: no explanatory variables (gray solid line), 10 variables from our regression analyses (black dash-dotted line), 10 variables plus country fixed effects (gray dashed line), 10 variables plus cluster fixed effects (black dashed line), and 10 variables plus mother fixed effects (black solid line)

So far, we have shown that age profiles differ by regions and by individual characteristics of the child, mother, or household (Figs. 3–5), and we have quantified these differences using within-mother variation (multivariate regression analyses in the Regression Model and Results sections). In this section, we combine these approaches in a multivariate graphical analysis (see Fig. 6). We first graph age curves without any covariates (gray solid line) and then add variables from our regression analyses as controls (black dash-dotted line), country fixed effects (gray dashed line), cluster fixed effects (black dashed line), and finally mother fixed effects (black solid line).^23^ We always plot residuals obtained after netting out these factors.

The graphs can be interpreted under two aspects. First, one can look at changes in the shape of the curves when covariates and fixed effects are netted out. Second, one can examine shifts away or toward the zero line. The closer the curve is to the zero line (i.e., the smaller the residual), the better the model fits the data and explains age heterogeneities.

Figure 6 suggests that inclusion of basic explanatory variables and country fixed effects does not have major implications neither in terms of shape nor shifts of the curves, both for height- and weight-for-age. On the contrary, community—and even more so, mother fixed effects—models fare much better, in particular for height-for-age. Up to 45 months, the MFE model predicts age patterns best (by pushing the residual age curve toward the zero line). Because mother fixed effects include a broad array of factors (e.g., country, community, and maternal factors), the resulting reduction in residual variance is not surprising. However, it is noticeable that the many cluster fixed effects—which account for community or “village” unobservable characteristics and are hierarchically just above the mother level—seem to have only one-half of the predictive power compared with the MFE. One possible explanation is that mothers are the “factor” in the closest contact with the babies, and the actual impacts of, say, community factors are likely shaped, compensated, or reinforced by mothers and their behavior.

Last, note that while the dips and bends are less pronounced using within-mother variation, they do remain. What is more, we observe an *increase* in the MFE residual after 21 months. However, this increase cannot be undoubtedly attributed to “catch-up growth” among older age groups for two reasons. First, given that the age range in our sample is restricted to 0–5 years, the observed increase could simply be an artifact of the data. Observing age cohorts up to, say, 14 years would offer a more appropriate base for catch-up growth statements. Second, as Lundeen et al. (2014) pointed out, such patterns could also stem from higher variance of HAZ among older children.

## Discussion and Conclusion

Undernutrition and growth faltering still hamper the socioeconomic progress of many developing countries. Although the percentage of stunted children fell from 40 % in 1990 to 27 % in 2010 across the globe, most of this decrease is reportedly driven by improvements in Asia. By contrast, the proportion of stunted children in Africa has been stagnating at approximately 40 %; and because of the persistent population growth there, the absolute number of stunted children has been actually increasing (see de Onis et al. 2012). Our article underlines that apart from being widespread, stunting varies across age groups in *all* developing regions, not only in Africa.

The original study by Shrimpton et al. (2001) on growth faltering sparked a focus on critical windows of child development. Height and weight are relatively low among many of the young children in our sample, pointing to substantial growth faltering. Our contribution to the discussion on undernutrition consists mainly in identifying age-specific correlates of child growth with more rigorous estimation methods than was done in the past. In particular, we pinpoint a wide range of correlates in a large sample of developing countries using both nonparametric and fully age-interacted MFE models. Unlike some single-country studies (Sahn and Alderman 1997), we do find that, for instance, the correlation of maternal education is magnified among the older age groups. We conjecture that our MFE models with an extensive set of time trends can be suggestive of heterogeneous growth faltering in early childhood.

Understanding socioeconomic gradients in child growth can inform cost-benefit analysis, such as assessing the returns to maternal education in terms of average height-for-age. In a simple OLS model that does not take into account age interactions (results not shown), moving from no maternal education to primary education is associated with a general 0.164 increase in HAZ. Incorporating age heterogeneities, however, changes the picture (see the left panel, Table 2): the marginal effect of maternal education amounts to an increase in HAZ by 0.065, 0.125, 0.182, and 0.206 standard deviations for newborns, 12-, 24-, and 36-month-old children, respectively. To illustrate the possible impact of such an increase in HAZ, note that a long-term study in Guatemala by Hoddinott et al. (2011:33) showed that boosting child’s height-for-age by 1 standard deviation at the age of 36 months “raises the per capita consumption level of the household that they live in by nearly 20 percent” in adulthood.

What could theoretically explain some of the age heterogeneities that we observe *within* a family? Our main outcome, HAZ, is an indicator of child health that was accumulated over time (Rieger and Wagner 2015). And accumulation of health capital can slow down because of adverse socioeconomic conditions and chronic illness (Case et al. 2002). In this sense, if unfavorable conditions persist, they might have a magnifying negative impact on the accumulated “stock” of health, whereas favorable conditions might amplify the positive effects. For instance, maternal education may lead to better maternal behaviors and access to health and childcare (Caldwell and Caldwell 1993; Desai and Alva 1998) at each point of a child’s (early) life. These positive maternal impacts may add up over time, thus speeding up child development and the accumulation of health capital.

Our article also aims at finding the best empirical predictors of age heterogeneities in child nutrition (see the earlier section, Grouping Age-Specific Correlates of Child Growth). We show that even when mother fixed effects are accounted for, age interactions remain important covariates in child growth regressions. Furthermore, mother fixed effects (as opposed to, say, cluster fixed effects) are a good predictor of what has been coined the first “1,000 days” of child development. In particular, maternal characteristics seem to have the strongest influence up to 45 months, which is well beyond the often quoted two-year opportunity window.

Finally, we would like to discuss limitations and possible avenues for future research. First, it would be useful to apply our modeling approach to investigate undernutrition patterns among children older than 5 years. If children can recover from spells of early growth faltering, it is of interest to see the impacts of such a recovery. As Crookston et al. (2013:1555) noted, a boost in “child growth after early faltering might have significant benefits on schooling and cognitive achievement.” Second, we have exclusively focused on height**-** and weight-for-age *z* scores. Other measures of health may yield different insights. For instance, Cameron and Williams (2009) detected no significant age heterogeneities in the correlation between household income and self-reported health for Indonesian children between 0 and 14 years. Third, it would be interesting to examine whether actual investment patterns of parents (e.g., nutritional inputs, medical care, hygiene, quality of water, parental time allocation) as a function of their child’s age are in line with the child growth age profiles documented in this article. Fourth, one could consider also other relevant factors, such as child’s medical history or chronic illnesses, which are unfortunately not available in our data. Fifth, in the absence of comparable panel data for a large number of countries, we had to rely on cross-sectional data. Despite our new modeling approach based on within-mother variation, our analysis is still limited to age-specific correlates of child growth. Panel data, on the other hand, would allow studying the actual “drivers” of growth faltering. Future research could contrast and cross-validate panel and cross-sectional analyses in countries where both are available. Related to this, it would be also interesting to investigate how the age profiles of child anthropometrics vary *within* a given region or country over time—in particular, following periods of rapid economic growth.

## Electronic supplementary material

ESM 1 (DOCX 28.5 kb)

## Footnotes

1 Throughout this article, we use terms height-for-age, height-for-age *z* score, and HAZ interchangeably; we always refer to height-for-age *z* scores.

2 See, for instance, the 1,000 Days website (www.thousanddays.org).

3 We study both height and weight because HAZ and WAZ tend to follow different patterns over time. Thus, the drivers of their faltering might also differ (Maleta et al. 2003).

4 Previous studies that have explored age heterogeneities have simply split the sample by age groups (Sahn and Alderman 1997).

5 Income growth seems to have similar impact independently of whether measured at the country or household level (Haddad et al. 2003).

6 Although Fernald et al. (2011) did include household effects, they used random rather than fixed effects. In our case, Hausman test rejects consistency of random-effects model, meaning that the fixed-effects model is more appropriate (*p* value = .000).

7 Histograms are available on request from the authors.

8 Wealth is measured by an asset index available in the DHS data. The index is constructed separately for each survey using a principal component analysis. Based on this index, households in each survey are ranked into five quintiles. We measure access to sanitation and to health centers at the cluster level. We define access to health center as a mother who visited a health center in the last 12 months.

9 Under-5 mortality is calculated directly by the DHS using a synthetic cohort life-table approach covering five years preceding the survey. Data on availability of calories per day per capita is taken from the Food and Agriculture Organization (FAO) of the United Nations. The drought variable comes from the International Disaster Database (version v12.07) available online (www.emdat.be). We take World Bank data on GDP per capita in constant 2011 international dollars, adjusted for differences in purchasing power parity (PPP) across countries. Because age of children is measured in months and GDP data are available only on an annual basis, we create a monthly measure of GDP through linear interpolation of annual GDP data.

10 Panel data would allow identifying growth-faltering patterns by following individuals over time and accounting more fully for child-specific heterogeneity or mortality shocks.

11 The mother fixed-effects sample is slightly more homogenous in terms of mother’s age because it covers women in their most productive childbearing ages (20–29 years) with a somewhat higher probability; the number of children decreases to one-half, and the number of mothers decreases to one-third in this reduced sample.

12 All figures present a local polynomial smooth of degree three of HAZ and WAZ, mostly with 95 % confidence bands (dashed lines). The confidence bands are generally tight given the large sample size. Thus, we suggest focusing on “economic significance” in addition. Local polynomial smooth corresponds to plotting means smoothed by local polynomial regression (*lpoly* in STATA 13). For the sake of comparison, we also plotted simple means by age groups as in Victora et al. (2010) and medians; the results were very similar.

13 We define structural break as the age with the lowest weighted average value of HAZ.

14 One methodological implication of these regional differences for later regression analyses is that we need to estimate region-specific age thresholds for HAZ and allow the nutrition-age profiles to vary by region.

15 When we split the regional age curves by birth order, we find that there are few differences between firstborn and later-born children in SSA, EAP, and SA excluding India. In India, however, the age profile shows a substantially bigger dip for later-born children. Additionally, the age curve of firstborn Indians is similar to that of African children, thus confirming the findings of Jayachandran and Pande (2015). Furthermore, we find that HAZ at birth does not significantly differ among SSA, SA, and India; in EAP, however, it is by approximately 1 standard deviation *higher*. On the other hand, mothers in SSA are 6cm taller than Asian mothers, and there is no important difference within Asia. Thus, we see that babies initially have similar length and height, and differences emerge only for older children and adults.

16 Region-specific structural breaks were estimated as follows: 18 (SSA region), 21 (MENA, SA regions), 22 (EAP region), 23 (LAC region), and 35 (ECA region) months of life.

17 Of the 17 candidate variables, the following seven did not fulfill these criteria: mother’s parity, mother suffered any child deaths, maternal literacy, difference in parental education, household size, access to electricity, and access to sanitation (cluster level). Variables shown only in subsample analyses because of data unavailability in several countries are preceding birth interval, breast-feeding, maternal smoking, access to health centers (cluster level), and calories availability (country level).

18 If children have the same father, then the mother fixed effect implicitly controls also for the father fixed effect. In the Indian data, for instance, only 0.03 % of children in the MFE sample have different fathers. Because this is an extremely low fraction, children with different fathers are unlikely to drive or contaminate our main results.

19 The model for weight-for-age is specified with a logarithmic function of age instead of the structural break, as discussed in the Data section. Additionally, an alternative specification of WAZ as a quadratic function of age is estimated as a robustness check in Table S2 in Online Resource 1.

20 The MFE model is estimated on deviations from maternal means, which means that the coefficients are identified by comparing siblings. As a result, we cannot estimate the direct effect of maternal, household, and country characteristics on child height.

21 In order to check whether these results are not driven by the choice of (linear) functional form, we estimate also a model in region-specific quadratic form. The results are qualitatively similar and quantitatively larger: the effect of better maternal education is 0.054 for a 6-month-old and 0.115 for a 12-month-old baby.

22 HAZ results are qualitatively similar and available in Online Resource 1, Table S3.

23 Given that local polynomial smooths cannot be estimated while controlling for additional covariates in STATA, we take a different approach. First, we regress HAZ and WAZ on a set of variables. Then we save residuals from this regression and graph them with a local polynomial smooth in Fig. 6.
