# Supplementary material for: Age-Specific Correlates of Child Growth
Source: Demography. 2016 Jan 4;53(1):241–67. doi: 10.1007/s13524-015-0449-3 (PMC4740575; doi:10.1007/s13524-015-0449-3)
Supplement: Supplementary file 1 — (DOCX 28.5 kb) [file 13524_2015_449_MOESM1_ESM.docx]

**Online Resource 1**

| Table S1: Nationally representative DHS surveys included in the analysis | | | | |
| --- | --- | --- | --- | --- |
| Country | Region | Survey  Year | Number of Mothers | Number of Children |
| Albania | ECA | 2008-09 | 1,283 | 1,524 |
| Armenia | ECA | 2010 | 1,126 | 1,414 |
| Azerbaijan | ECA | 2006 | 1,585 | 2,090 |
| Bangladesh | SA | 2011 | 6,890 | 7,951 |
| Benin | SSA | 2011-12 | 8,304 | 11,539 |
| Bolivia | LAC | 2008 | 6,061 | 7,854 |
| Burkina Faso | SSA | 2010 | 4,961 | 6,736 |
| Burundi | SSA | 2010-11 | 2,346 | 3,504 |
| Cambodia | EAP | 2010-11 | 3,101 | 3,824 |
| Cameroon | SSA | 2011 | 3,633 | 5,195 |
| Chad | SSA | 2004 | 3,202 | 4,679 |
| Colombia | LAC | 2009-10 | 13,374 | 16,055 |
| Congo | SSA | 2011-12 | 3,290 | 4,535 |
| Cote d'Ivoire | SSA | 2011-12 | 2,477 | 3,306 |
| Democratic Republic of the Congo | SSA | 2007 | 2,482 | 3,696 |
| Dominican Republic | LAC | 2007 | 7,559 | 9,540 |
| Egypt | MENA | 2008 | 7,859 | 10,471 |
| Ethiopia | SSA | 2011 | 7,068 | 9,941 |
| Gabon | SSA | 2012 | 2,550 | 3,504 |
| Ghana | SSA | 2008 | 1,951 | 2,559 |
| Guinea | SSA | 2012 | 2,427 | 3,215 |
| Guyana | LAC | 2009 | 1,325 | 1,745 |
| Haiti | LAC | 2012 | 3,190 | 4,046 |
| Honduras | LAC | 2011-12 | 8,236 | 10,049 |
| India | SA | 2005-06 | 32,963 | 43,736 |
| Jordan | MENA | 2012 | 4,286 | 6,386 |
| Kenya | SSA | 2008-09 | 3,774 | 5,367 |
| Kyrgyz Republic | ECA | 2012 | 3,037 | 4,096 |
| Lesotho | SSA | 2009-10 | 1,372 | 1,673 |
| Liberia | SSA | 2006-07 | 3,464 | 4,592 |
| Madagascar | SSA | 2008-09 | 3,737 | 5,043 |
| Malawi | SSA | 2010 | 3,587 | 4,911 |
| Maldives | SA | 2009 | 2,378 | 2,693 |
| Mali | SSA | 2006 | 8,243 | 11,641 |
| Moldova | ECA | 2005 | 1,245 | 1,392 |
| Morocco | MENA | 2003-04 | 4,515 | 5,681 |
| Mozambique | SSA | 2011 | 7,034 | 9,719 |
| Namibia | SSA | 2006-07 | 3,172 | 3,865 |
| Nepal | SA | 2011 | 1,892 | 2,363 |
| Nicaragua | LAC | 2001 | 4,686 | 6,180 |
| Niger | SSA | 2012 | 3,385 | 5,161 |
| Nigeria | SSA | 2008 | 16,063 | 23,075 |
| Pakistan | SA | 2012-13 | 2,461 | 3,636 |
| Peru | LAC | 2009 | 7,884 | 9,442 |
| Rwanda | SSA | 2010-11 | 3,068 | 4,128 |
| Sao Tome and Principe | SSA | 2008-09 | 1,328 | 1,708 |
| Senegal | SSA | 2010-11 | 2,805 | 3,937 |
| Sierra Leone | SSA | 2008 | 1,766 | 2,279 |
| Swaziland | SSA | 2006-07 | 1,700 | 2,112 |
| Tajikistan | ECA | 2012 | 3,397 | 4,771 |
| Tanzania | SSA | 2009-10 | 4,954 | 6,969 |
| Timor-Leste | EAP | 2009-10 | 5,571 | 8,477 |
| Turkey | ECA | 2003-04 | 3,177 | 4,163 |
| Uganda | SSA | 2011 | 1,418 | 2,116 |
| Zambia | SSA | 2007 | 3,772 | 5,429 |
| Zimbabwe | SSA | 2010-11 | 3,716 | 4,439 |
| Total |  | 2001-2013 | 262,130 | 350,152 |
| **Note:** Regions are labelled as follows: Europe and Central Asia (ECA), East Asia and Pacific (EPA), Latin America and the Caribbean (LAC), Middle East and North Africa (MENA), South Asia (SA), Sub-Saharan Africa (SSA). | | | | |

| Table S2: Robustness check - Alternative regression results for weight-for-age (WAZ) | | | | | | |
| --- | --- | --- | --- | --- | --- | --- |
| Specification | OLS | | | MFE | | |
| Coefficient Variable | Variable | Age *  Variable | Age sq.* Variable | Variable | Age *  Variable | Age sq.* Variable |
| Child is a girl * | 0.080* | 0.004* | -0.149* | 0.086* | 0.004 | -0.139* |
|  | (0.027) | (0.002) | (0.000) | (0.042) | (0.003) | (0.000) |
| Child's birth order | 0.010 | -0.002* | 0.029* | -0.643* | -0.001+ | 0.021+ |
|  | (0.007) | (0.000) | (0.000) | (0.030) | (0.001) | (0.000) |
| Mother's height (in 10 cm) | 0.399* | -0.005* | 0.063* | - | 0.005* | -0.081* |
|  | (0.020) | (0.002) | (0.000) |  | (0.002) | (0.000) |
| Mother's age at marriage | 0.006 | 0.001* | -0.011* | - | 0.001+ | -0.009 |
|  | (0.004) | (0.000) | (0.000) |  | (0.000) | (0.000) |
| Mother's education (in levels) | 0.151* | 0.003* | -0.044* | - | 0.009* | -0.134* |
|  | (0.018) | (0.001) | (0.000) |  | (0.002) | (0.000) |
| Poorest quintile * | -0.076* | -0.012* | 0.207* | - | -0.008* | 0.158* |
|  | (0.037) | (0.003) | (0.000) |  | (0.004) | (0.000) |
| Rural area * | -0.136* | -0.001 | 0.016 | - | -0.000 | -0.017 |
|  | (0.034) | (0.002) | (0.000) |  | (0.003) | (0.000) |
| Drought * | 0.486* | -0.032* | 0.449* | - | 0.002 | 0.278* |
|  | (0.042) | (0.003) | (0.000) |  | (0.007) | (0.000) |
| Under-5 mortality (per 100 births) | 0.007+ | -0.003* | 0.052* | - | -0.004* | 0.015+ |
|  | (0.004) | (0.000) | (0.000) |  | (0.001) | (0.000) |
| GDP p.c. PPP (in 1000$) | 0.067* | -0.002* | 0.019* | - | 0.004* | -0.046* |
|  | (0.005) | (0.000) | (0.000) |  | (0.001) | (0.000) |
| Observations | 316,389 | | | 316,389 | | |
| Observations contributing to variance | 316,389 | | | 155,014 | | |
| R-squared | 0.247 | | | 0.117 | | |
| **Notes:** OLS and mother fixed effects (MFE) estimations. Dependent variable is z-score of weight-for-age. All explanatory variables marked with an asterisk are binary. Mother's education is measured in four levels: no education, primary, secondary, and higher education. Country level variables drought, under-5 mortality, and GDP are measured in the year of the survey. Coefficients of age, age squared, their interactions with regional dummy variables (to allow for the main WAZ-age profile to vary by region), year-of-survey fixed effects, and a constant are estimated but not shown. MFE specification includes also country-specific time trends, year-of-birth fixed effects, calendar-month-of-birth fixed effects, and difference-between-calendar-month-of-survey-and-calendar-month-of-birth fixed effects. **Coefficients and standard errors of "age squared * variable" are multiplied by 1,000.** Coefficients of variables that do not vary among siblings cannot be estimated in MFE specification. Regressions are weighted using population-size-adjusted sampling weights. Standard errors are clustered at the primary sampling unit (cluster) level and are shown in parentheses. Significance levels are marked as follows: * p<0.05, + p<0.1. | | | | | | |

| Table S3: Robustness check - OLS estimation on MFE sample | | | | | | |
| --- | --- | --- | --- | --- | --- | --- |
| Dependent variable | Height-for-age (HAZ) | | | Weight-for-age (WAZ) | | |
| Coefficient Variable | Variable | Age *  Variable | Break * Variable | Variable | Age *  Variable | Age sq.* Variable |
| Child is a girl * | 0.124* | 0.001 | 0.214* | 0.062 | 0.007* | -0.202* |
|  | (0.055) | (0.004) | (0.089) | (0.038) | (0.003) | (0.000) |
| Child's birth order | 0.007 | -0.002* | -0.010 | 0.020* | -0.004* | 0.064* |
|  | (0.014) | (0.001) | (0.021) | (0.010) | (0.001) | (0.000) |
| Mother's height (in 10 cm) | 0.400* | 0.006+ | 0.043 | 0.364* | -0.003 | 0.033 |
|  | (0.039) | (0.003) | (0.065) | (0.028) | (0.002) | (0.000) |
| Mother's age at marriage | -0.002 | 0.001 | 0.009 | 0.012* | -0.000 | 0.005 |
|  | (0.008) | (0.001) | (0.012) | (0.005) | (0.000) | (0.000) |
| Mother's education (in levels) | 0.040 | 0.005+ | 0.058 | 0.141* | 0.003 | -0.040 |
|  | (0.036) | (0.003) | (0.056) | (0.025) | (0.002) | (0.000) |
| Poorest quintile * | -0.048 | -0.013* | -0.142 | -0.099* | -0.011* | 0.189* |
|  | (0.069) | (0.005) | (0.107) | (0.049) | (0.004) | (0.000) |
| Rural area * | -0.056 | -0.005 | -0.193+ | -0.129* | 0.001 | -0.027 |
|  | (0.066) | (0.005) | (0.101) | (0.048) | (0.003) | (0.000) |
| Drought * | 0.371* | -0.022* | -0.460* | 0.475* | -0.029* | 0.402* |
|  | (0.086) | (0.007) | (0.137) | (0.057) | (0.004) | (0.000) |
| Under-5 mortality (per 100 births) | 0.010 | -0.003* | -0.006 | 0.015* | -0.003* | 0.050* |
|  | (0.007) | (0.001) | (0.012) | (0.005) | (0.000) | (0.000) |
| GDP p.c. PPP (in 1000$) | -0.000 | 0.002* | 0.065* | 0.074* | -0.001* | 0.012 |
|  | (0.008) | (0.000) | (0.021) | (0.007) | (0.001) | (0.000) |
| Observations | 305,967 | | | 316,389 | | |
| Observations contributing to variance | 144,432 | | | 155,014 | | |
| R-squared | 0.179 | | | 0.226 | | |
| **Notes:** OLS estimation. Model specification is identical to OLS regressions shown in Table 2 and S2 but the sample is restricted to observations that contribute to variation in the mother fixed effects (MFE) estimation. **Coefficients and standard errors of "age squared * variable" are multiplied by 1,000.** Standard errors are clustered at the primary sampling unit (cluster) level and are shown in parentheses. Significance levels are marked as follows: * p<0.05, + p<0.1. | | | | | | |

| Table S4: Additional analyses on subsamples for height-for-age (HAZ) | | | | | | |
| --- | --- | --- | --- | --- | --- | --- |
| Specification | OLS | | | MFE | | |
| Coefficient Variable | Variable | Age *  Variable | Break * Variable | Variable | Age *  Variable | Break * Variable |
| Birth interval (in years) | 0.014 | 0.002* | 0.037* | -0.280* | 0.012* | 0.282* |
|  | (0.010) | (0.001) | (0.018) | (0.065) | (0.003) | (0.073) |
| Observations | 231,186 | | | 231,186 | | |
| Observations contributing to variance | 231,186 | | | 100,959 | | |
| R-squared | 0.175 | | | 0.262 | | |
| Breastfed at least 12 months * | 0.044 | -0.007 | -0.333* | 0.257 | -0.005 | -0.358 |
|  | (0.087) | (0.005) | (0.122) | (0.208) | (0.012) | (0.237) |
| Observations | 195,742 | | | 195,742 | | |
| Observations contributing to variance | 195,742 | | | 84,908 | | |
| R-squared | 0.176 | | | 0.247 | | |
| Mother smokes * | -0.298* | 0.016* | 0.192 | - | 0.017+ | 0.307 |
|  | (0.091) | (0.006) | (0.139) |  | (0.009) | (0.213) |
| Observations | 259,904 | | | 259,904 | | |
| Observations contributing to variance | 259,904 | | | 126,420 | | |
| R-squared | 0.178 | | | 0.252 | | |
| Visited health center (cluster level) | -0.099 | 0.012 | -0.121 | - | -0.005 | -0.025 |
|  | (0.099) | (0.008) | (0.142) |  | (0.012) | (0.202) |
| Observations | 253,284 | | | 253,284 | | |
| Observations contributing to variance | 253,284 | | | 122,243 | | |
| R-squared | 0.145 | | | 0.219 | | |
| Calories (in 1000 kcal p.c. per day) | -1.748* | 0.108* | 2.911* | - | 0.028 | 1.854+ |
|  | (0.679) | (0.047) | (0.669) |  | (0.102) | (1.057) |
| Observations | 94,218 | | | 94,218 | | |
| Observations contributing to variance | 94,218 | | | 44,367 | | |
| R-squared | 0.162 | | | 0.252 | | |
| **Notes:** OLS and mother fixed effects (MFE) estimations. Dependent variable is z-score of height-for-age. All explanatory variables marked with an asterisk are binary. Variable "visited health center" corresponds to fraction of women who visited a health center in the last 12 months, measured at the cluster level. Country level variable "calories" measures calorie supply per capita in each country for calories available from crop products. Each panel corresponds to regressions identical to those in Table 2 ("base" model); the displayed variable and its corresponding interactions are added to the "base" model. Standard errors are clustered at the primary sampling unit (cluster) level and are shown in parentheses. Significance levels are marked as follows: * p<0.05, + p<0.1. | | | | | | |

| Table S5: Additional analyses on subsamples for weight-for-age (WAZ) | | | | |
| --- | --- | --- | --- | --- |
| Specification | OLS | | MFE | |
| Coefficient Variable | Variable | Log(Age) *  Variable | Variable | Log(Age) *  Variable |
| Birth interval (in years) | 0.018+ | 0.005 | -0.030 | 0.020 |
|  | (0.010) | (0.003) | (0.055) | (0.014) |
| Observations | 239,267 | | 239,267 | |
| Observations contributing to variance | 239,267 | | 108,585 | |
| R-squared | 0.246 | | 0.148 | |
| Breastfed at least 12 months * | 0.052 | -0.048* | 0.017 | 0.004 |
|  | (0.084) | (0.024) | (0.166) | (0.045) |
| Observations | 201,046 | | 201,046 | |
| Observations contributing to variance | 201,046 | | 90,641 | |
| R-squared | 0.271 | | 0.128 | |
| Mother smokes * | -0.419* | 0.112* | - | 0.082* |
|  | (0.088) | (0.026) |  | (0.035) |
| Observations | 268,717 | | 268,717 | |
| Observations contributing to variance | 268,717 | | 135,806 | |
| R-squared | 0.230 | | 0.129 | |
| Visited health center (cluster level) | -0.490* | 0.208* | - | 0.093* |
|  | (0.100) | (0.030) |  | (0.041) |
| Observations | 261,694 | | 261,694 | |
| Observations contributing to variance | 261,694 | | 131,280 | |
| R-squared | 0.165 | | 0.132 | |
| Calories (in 1000 kcal p.c. per day) | 15.302* | 0.272* | - | 0.451 |
|  | (1.748) | (0.122) |  | (0.456) |
| Observations | 94,814 | | 94,814 | |
| Observations contributing to variance | 94,814 | | 46,617 | |
| R-squared | 0.199 | | 0.129 | |
| **Notes:** OLS and mother fixed effects (MFE) estimations. Dependent variable is z-score of weight-for-age. All explanatory variables marked with an asterisk are binary. Variable "visited health center" corresponds to fraction of women who visited a health center in the last 12 months, measured at the cluster level. Country level variable "calories" measures calorie supply per capita in each country for calories available from crop products. Each panel corresponds to regressions identical to those in Table 3 ("base" model); the displayed variable and its corresponding interactions are added to the "base" model. Standard errors are clustered at the primary sampling unit (cluster) level and are shown in parentheses. Significance levels are marked as follows: * p<0.05, + p<0.1. | | | | |
